# Through Internet and Friends: Translation of Air Pollution Research in Malmö Municipality, Sweden

**DOI:** 10.3390/ijerph17124214

**Published:** 2020-06-12

**Authors:** Ebba Lisberg Jensen, Karin Westerberg, Ebba Malmqvist, Anna Oudin

**Affiliations:** 1 Urban Studies, Malmö University, 205 06 Malmö, Sweden; karin@akademiskskrivkonst.com; 2 Occupational and Environmental Medicine, Department for Laboratory Medicine, Lund University, Tornblad Institute, Biskopsgatan 9, 223 62 Lund, Sweden; ebba.malmqvist@med.lu.se (E.M.); anna.oudin@med.lu.se (A.O.); 3 Occupational and Environmental Medicine, Department Public Health and Clinical Medicine, Umeå University, 901 87 Umeå, Sweden

**Keywords:** air pollution abatement, public health, research knowledge translation, sustainable urban planning, municipal civil servants, sustainable urban development, knowledge access practices, collaborative research

## Abstract

Air pollution is estimated to cause more than 7000 deaths annually in Sweden alone. To reduce the impact of air pollution and to plan and build sustainable cities, it is vital that research is translated into efficient decisions and practice. However, how do civil servants in a municipality access research results? How do they normally find relevant information, and what obstacles are there to accessing and applying research results? As part of the collaborative and transdisciplinary research project Air Pollution Research in Local Environmental Planning (ARIEL), these questions were explored through interviews and seminars with civil servants within the Malmö Municipality Environmental Office. We found that the civil servants generally have proficiency in processing research results, but often do not use such results as part of their everyday decision making and practices. Instead, the data and measurements used are mostly produced case-by-case within the municipal sector itself. Information about best practices is also collected via a number of knowledge access practices, involving the Internet or social networks within other municipalities. Lack of time, paywalls, and the insufficient applicability of research hinder the dissemination of up-to-date results. This slows down the process whereby research, funded by tax-money, can be put to best practice in the effort to create healthy and sustainable cities.

## 1. Introduction

**Figure 1:**
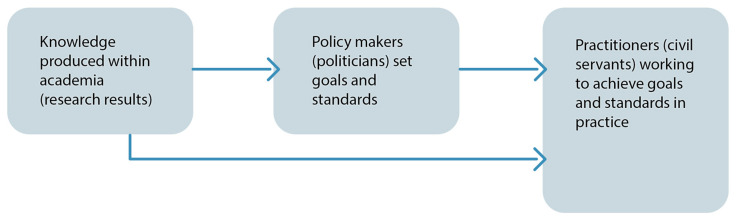
Ideal model of how research results are expected to be applied in policy making and practice in the Swedish political and administrative context.

Research on air pollution is produced to improve the living conditions of citizens. Still, in Sweden, a country with traditionally high set standards for environmental research [1] as well as public health policy, air pollution is estimated to cause more than 7000 premature deaths annually [2]. Traffic-related air pollution has been linked to respiratory and cardiovascular diseases [3], as well as negative effects on foetal growth [4] and cognition [5], all leading to human suffering and costs for society. Exposure to air pollution affects socioeconomically weaker groups more, and can also be seen as an issue of environmental inequality [6]. To live up to the UN Sustainability Goals 2030, especially goal 3, good health and wellbeing; goal 10, reduced inequality; goal 11, sustainable cities and communities [7]; and goal 13, climate action, cities need to address air pollution by taking necessary action based on robust research [8]. In Sweden, tax money is used to finance research on how to achieve sustainable urban development. Policy makers are expected to base policy formulation on research results so as to set goals and standards (Figure 1). The policy then is supposed to be put into best practice by civil servants (*tjänstemän*), who, in the Swedish context, are non-politically appointed employees in municipal or national administration. Their job is to translate policy into practice, ideally informed by research results for optimal outcomes. At a first glance, the process from knowing to deciding to doing looks uncomplicated (Figure 1). However, the last step, when research is translated into practice, is not always as straightforward as might be expected. 

**Figure 2:**
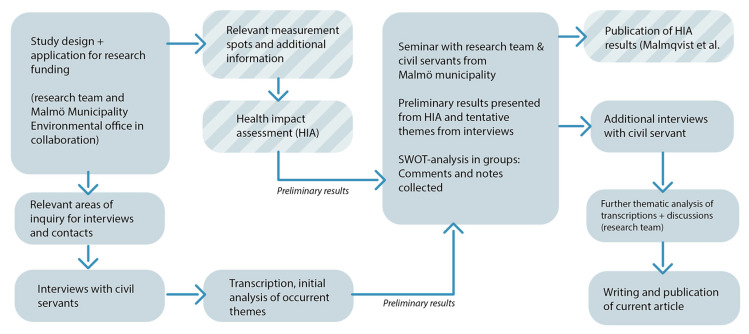
Flowchart of collaborative and transdisciplinary research project Air Pollution Research in Local Environmental Planning (ARIEL), from the design of quantitative and qualitative strands of research via studies and feedback seminars to publications.

To explore and facilitate more scientifically based practices in building denser cities, without causing increased negative health impacts from air pollution, we initiated a collaborative and transdisciplinary research project called Air Pollution Research in Local Environmental Planning (ARIEL) (see Figure 2). The city of Malmö, southern Sweden, was chosen as the study area, since it has had problems complying with the EU Directive 2008/50/EC and WHO Air Quality Guidelines of 40 µg/m^3^ NO_2_ as an annual mean in the past, and has had action plans since 2006 to reduce emissions. The city has reduced emissions and complied with these regulations since 2016. The annual means of NO_2_ in Malmö 2018–2019 were 10–12 µg/m^3^ at the urban background site at the roof level, and 19–26 µg/m^3^ at traffic sites, thus sometimes exceeding the Swedish Environmental Objective for clean air, which for NO_2_ is 20 µg/m^3^ as annual mean. The annual means of PM_2.5_ in Malmö were 9 µg/m^3^ in urban background sites and 10–13 µg/m^3^ in traffic sites during the 2018–2019 period. This means that Malmö complies with the EU Directive 2008/50/EC of annual means of 25 µg/m^3^, but not always with the more health-related WHO air quality guidelines or Swedish Environmental Objectives, both with an annual mean of 10 µg/m^3^ [9,10].

An overarching aim of the ARIEL project was to create a recursive knowledge flow between researchers and municipal civil servants to improve knowledge and the applicability of research results [11] and facilitate better practices in air pollution abatement [12]. Scholars from Malmö, Lund, and Umeå Universities collaborated with the Malmö Municipality Environmental Office in designing the study and formulating the research application. Two strands of study were decided upon, one quantitative and one qualitative (see Figure 2). The municipality office provided relevant measuring points and earlier data for the quantitative study related to air pollution. Knowledge translation was discussed from the practitioners’ standpoint, and the areas of inquiry were outlined for the qualitative interview study. It was decided to also include civil servants working with noise abatement, since the two issues were related in practice. A seminar for researchers and civil servants was also planned so that the preliminary results could be discussed between the two groups and the results returned to the practitioners (see Figure 1).

The quantitative part of the project was a health impact assessment, HIA. The models of two traffic scenarios in Malmö were outlined in discussions with the Environmental Office. The HIA also estimated the societal costs of pollution and of lowered costs when mitigating hazards. One of our findings was that up to 4% of all premature deaths could be prevented if all petrol- and diesel-powered vehicles were banned from Malmö [13]. The results of the HIA were published in Malmqvist et al. [13] and spread in popular forms in an information folder produced in collaboration between the research team and the Environmental Office [14] and a Youtube video, entitled “The health effect of air pollution from traffic” [15]. 

The qualitative part of the project presented here focuses on how civil servants, working with air pollution and noise abatement in Malmö municipality, access, process, and apply research results and other sources of information in their everyday work. Studying this step of the translation process aims at a better understanding of the realities of translation between academia and civil servants. What relations to research form the basis of their professional lives? When faced with complex planning issues, how do they go about finding necessary information, and what role do scientific results play in this process? What are the possibilities for employees in the municipal office for accessing, processing, and applying scientific information in their practical work? How do new research results reach them for optimal implementation? What are the obstacles to research results reaching municipal civil servants in Malmö municipality?

We start with a brief overview of previous research regarding the translation of knowledge from research to practice, which takes us into fields such as health science, education, and linguistics. We then present the methods applied in the qualitative phase of this research project and our findings from the interview study.

### Knowledge Translation in Previous Research 

As a foundation for building a sustainable and healthy society, research is supposed to provide authorities with the relevant knowledge, so that politicians can make evidence-based decisions and civil servants can implement them in the most rational way. However, Jasanoff early on pointed to a naïveté regarding the capacity of science to analyse and solve environmental problems [16]. The “linear development”, where scientific knowledge is expected to gradually dissolve into society, is often slowed down by social complications [17]. The complexity of methodology and the uncertainty of results may complicate the translation process. Among scientists themselves, there is an understanding that measurements to some extent must be tentative, but it is the job of the researcher to catch and address errors and refine measurements [18]. Air pollution abatement is politically and administratively complex since the sources are mobile, as is the air, and there is also a need for the public understanding and acceptance of the measures taken to address the issues [19]. Rose et al. argue that for scientific results on environmental matters to be swiftly applied in larger society, scientists must be able to foresee what emergent environmental problems are going to be urgent in the public debate and legislation process [20]. During what they call the “policy window”, scientists need to be ready to package results for accessibility, frame them for best understanding in an era of information overload, and then persevere in efforts to put their results to best use by processes of communication. Most scholars in the field of knowledge translation focus on how scientific knowledge is translated to and understood by policy makers or legislators [17,21,22,23] and, in some cases, the public [24,25]. As many environmental issues are politically contested, translation directed to policy makers is of course pivotal. In our case though, the focus is to investigate how research results are accessed and applied by practitioners, i.e., the civil servants in Malmö municipality, assigned to achieve the already politically decided goals regarding air pollution and, in relevant cases, noise abatement.

Research on knowledge translation to practitioners has previously focused on other areas than environmental issues. Ginsburg and Gorostiaga investigated knowledge utilisation within the field of education. They suggested models for transmission of knowledge that might be applicable also to the environmental arena. Their first, “knowledge-driven” model is of relevance for how research in air pollution abatement is supposed to work. Shortly, basic research is expected to lead to applied research, which should lead to development and application [26]. The assumption is that “good science will trickle down to the level of practice and inform practitioners on what to do and not to do” [26]. Within the field of medicine and health-care, where it is pivotal that best practice is utilised without delay, understanding how to optimise the process has been named knowledge translation research. According to Curran et al. [27], knowledge translation is “the process of moving from what has been learned through research to application in different decision-making contexts” [27]. “Decisions” can be understood as what it is decided to actually do—that is, the praxis related to research. Graham et al. name this part of the process knowledge transfer [28]. Still, there are many stages where translation and transfer might be blurred. Weiss [29] demonstrated that in the realm of social sciences, research, rather than being consciously or systematically utilised as a basis for decision-making, had a tendency to creep into the realms of policy and then eventually into practice to accrete there. Decisions were not consciously made, or at least did not follow formalised paths. Instead, Weiss stated, staff tended to not perceive their profession as transferring research to practice, but more as “doing their job” [29]. Decisions were seen as part of practice, and staff based “their ongoing actions on the sum of their knowledge and judgement” [29]. She also drew the conclusion that employees often did not store or catalogue research as a specific category in their minds, but merged scientific information with what they already knew and how they worked [29]. 

This rhymes with the findings of an investigation of civil servants in the Swedish Environmental Protection Agency (SEPA) [1]. The SEPA has a tradition of strong ties to academia. It employs researchers as civil servants and has its own research department. Studying how scientific knowledge was accessed and put to use by civil servants in the bureau in the 1980s, Lundgren and Sundqvist found that knowledge took differing forms depending on where in the process the civil servants accessed and applied it. Their suggested categories of knowledge among their interviewees were facts, understanding, proficiency, and familiarity [30], where research results occurred mainly in the first category. Among the civil servants, there was a tendency to not to define what kind of knowledge was utilised. It was not important to them, as long as they deemed the knowledge to be “scientifically acceptable” [30], and their way of accessing knowledge was reported to be sufficient for performing their task. The challenge was to understand how civil servants moved from theoretical to practical aspects of knowledge, from “knowing to doing” [30], and also to understand how research results could be better disseminated. Though science was held in high esteem among the civil servants at the SEPA, Lundgren and Sundqvist deducted that knowledge was mainly communicated, translated, and established within social networks rather than through formalised dissemination channels like publications, libraries, and reports. Despite the fact that Lundgren’s and Sundqvist’s study [30] was conducted more than three decades ago, their results strongly resonate with our findings, as we will see below. 

Human interactions function as organic channels of information in private life as well as professionally. Social networks, though, may have a tendency to conserve ideas and cognitively keep actors within the the realm of the already known. This knowledge-related path dependency is an effect of how humans generally exchange and disseminate ideas and practices. Milroy and Milroy argued, within the field of linguistics, that close-knit social networks enhance local cohesion and slow down the spread of new words [31]. Linguistic innovation, according to them, occurs more often in weak-tie networks, such as between casual acquaintances and colleagues. Their model is conceptually relevant here, where research results are the “language” in which arguments for certain actions are formulated. Ideally, in a modern administration, new and relevant research is expected not only to trickle but to flow into the realms of action among practitioners. However, as it happens, the way knowledge is spread among a group of professionals comes to resemble a conservative close-knit network more than the casual weak-tie network that Milroy and Milroy refers to as innovative and open to change. The professional network, thus, simultaneously functions as a channel for dissemination and slows down the spread of new information, such as research results. An important new factor in the circumstances of contemporary civil servants, as compared to those in Lundgren’s and Sundqvists’s study, is the digital revolution. It should, ideally, enable research results to flow to practitioners more easily than ever before. In the daily work of the civil servants studied here, though, there are a number of structural obstacles for direct access to up-to-date research, and they instead often rely on in-house information and social contacts when looking for relevant information. Despite the fact that the civil servants themselves report sufficient access to research results, a lack of direct access to research may slow down the dissemination of new results and methods. Before looking further into how this happens, we present how the study was designed and conducted.

## 2. Materials and Methods 

The qualitative part of the ARIEL project presented in this article (illustrated in the lower strand of Figure 1) focused on investigating how municipal civil servants in Malmö municipality engaged in air pollution abatement and connected noise-related issues access research results and other forms of knowledge to solve their respective tasks, in what ways they are processing the information acquired, and what obstacles there are for them to obtain and apply high-quality updated research. We performed semi-structured interviews with eight civil servants engaged in issues regarding air pollution abatement, traffic planning, and the Malmö municipality environmental plan [32], representing a purposive sample. An invitation to participate in a research interview was sent out via email by one of the leading civil servants in the Environmental Office to 11 employees whose work was considered relevant to the study, including a few who also worked with related noise abatement. It was made clear to the invited interviewees that participation was approved of as the municipality’s input of working hours into the collaborative research project. A letter from the researchers, explaining the aim of the study, was attached. After follow-up emails and phone-calls from the researchers, eight civil servants agreed to be interviewed. Two never responded despite intense follow-up and one had changed jobs. Out of the eight remaining, three were men and five were women. All of the interviewees held management positions within their working groups, and all had an academic education from disciplines like civil engineering, environmental science, environmental chemistry, political science, and physical geography.

In a qualitative thematic analysis like this, a small sample may provide just as much data saturation as a larger if the overarching research questions are met [33]. We detected a pattern in the material already after four interviews, and the pattern was clarified in the following four. With the purposive sample, we aimed to reach insights that are transferable and which, together with the given context and conceptual framework [34], contribute to a better understanding of how knowledge translation happens in a municipality and what obstacles it might meet.

Six of the semi-structured interviews were conducted during daytime in the Environmental Office and two were conducted in the Traffic Office. In addition to the initial invitation letter, author one, conducting the interviews, informed respondents that we were not aiming to evaluate their performance as individual employees and that we would not publish names, positions, or personal details, thus facilitating a more open conversation. We also clarified that the focus lay on their relation to and access to research, and on how research is applied in their daily work. The interviews, lasting from 45 to 60 min, were structured around questions designed to provide answers to the research aim. After the introductory questions about educational and professional history, we focused on the structural level, such as information and dissemination channels, formal and informal networks, and the time allowed for information gathering and processing, etc. During the interviews, the civil servants were encouraged to add other reflections that might refine our understanding. As is the case with research interviews, some respondents were talkative and others responded more sparsely, but all the interviews provided answers to the following questions:

- What education, previous types of employment and pre-knowledge do you have in your field, and how do you apply this in your professional life?
- Please describe how a project (in traffic- or environmental planning) arrives at your desk, how you start working on it and what obstacles or insecurities do you and your co-workers encounter when it comes to the effects of your actions…
- What kind of information do you normally rely on to carry out your projects in the most knowledgeable and efficient way?
- How do you access information regarding your work? What are your preferred channels of information? How do you trust your channels and evaluate them?
- Is there anything you miss out on when it comes to taking the best-informed decisions, or any channels for information you wish you and your office had access to?
- What obstacles do you encounter when looking for the latest information in relation to your work, and what do you wish could be organised differently for you to be more knowledgeable or well-informed when facing challenges in your work?

The interviews were recorded digitally and notes were taken during the interviews. The recordings were transcribed verbatim. The transcriptions were thoroughly read and coded into recurrent themes by the two first authors individually, who then compared notes and discussed the findings among the four authors. A second round of reading was carried out by the first two authors to mark relevant quotations. Already after four interviews, there was a clear pattern in the material, which became increasingly pervasive after the subsequent four interviews. Major themes, or categories, are presented in the finding section as subtitles. Minor categories, i.e., those that were relevant though not pervasive in the material, are included in the findings where they were found to be relevant.

The interviews were conducted in Swedish. For the purpose of this article, the relevant quotations were translated into English and lightly edited for better readability. Detailed personal information, like age, gender, and position, as well as the date of the interviews, has been omitted in the findings section to protect anonymity. 

In November 2018, an open seminar with civil servants from the Environmental Office, the Traffic Office, other interested civil servants in the Malmö municipality, the ARIEL research team, and a few external researchers was organised. During the seminar, a representative from the Environmental Office presented their current work on air pollution abatement, the standards met, and the remaining challenges. The results from the health impact analysis (HIA) were presented, as well as the tentative themes from the first four interviews with civil servants in the Malmö Environmental Office and Traffic Office. The 30 participants were then divided into groups to discuss their daily work in relation to knowledge access and to provide the research team with feedback on the tentative themes developed. The feedback was gathered in the form of notes created by each group and collected by the researchers. The final part of the seminar was a two-hour long group discussion between civil servants from the Environmental Office and the Traffic Office and the research team, organised as a SWOT-analysis (defining strengths, weaknesses, opportunities and threats) with focus on how to work more efficiently with air pollution abatement, and what sorts of knowledge and dissemination routines would be more efficient and applicable. The notes from this seminar were of help in the remaining interviews and also functioned as a possibility for the municipal civil servants to reflect constructively on their work. 

## 3. Findings and Discussions

How do civil servants working with air pollution and noise abatement in Malmö municipality access, process, and apply research results and other sources of information in their everyday work? In this section, we present each of the themes deduced from the transcriptions of the interviews, discussing them in their context and exemplifying them with quotations from the interviewed civil servants.

### 3.1. “Actually an Academic”—Personal Educational History

Swedish governmental and municipal administrations have a long tradition of hiring well-educated staff. “Hireability” is part of the academic planning of new educational programmes and is supposed to form a feedback loop, where professionals needed in society are provided by the educational system. According to one interviewee, civil servants in the municipality office are “very highly educated, very knowledgeable”. However, the acquired academic grades, pivotal when initially applying for a job in the administrative sector, might not be straightforwardly connected to the job an employee actually performs. Once inside the administration, formal education plays a smaller role and educational history may sometimes prove to be of less importance. In the early stages of a career, inexperienced young professionals cannot be picky about positions. Over time, they work themselves into professional networks, building up contacts and experience. As one respondent explained, her education within the natural sciences was fairly unrelated to her first position. Eventually, as she became more experienced, she was transferred into jobs more related to her education. Others reported long and winding roads to their present positions, sometimes in and out of the municipal sphere and the private sector. One respondent, untypically, reported an education as an engineer, where after he got a position in the municipality and stayed there for two decades. Another respondent had an education focused on public policy, which initially seemed to be only vaguely related to his position.

- I’m originally a political science student and took a masters’ degree, and I have an additional grade in environmental science. So, I’m an environmental science and policy graduate person.

- So, your first degree was more to the science side?

- No, it was an international master’s degree, a transdisciplinary one, but there were many scientific courses too. A lot of focus on system analysis. There was environmental economy and environmental law [too], and international environmental policy. So–a great variety. And then I graduated.

This respondent had worked in a row of functions regarding traffic, urban planning, logistics, and environmental norms, somewhat typical for the generalist background mentioned above (digressions omitted):

- I started working here in 2002, with traffic issues. It was an entrance to a position about environmentally friendly commuting […]. “Mobility management”, it was called back then. […] Then it turned increasingly into investigative work [*utredningsarbete*], developing the traffic environment programme. I became a project manager there […] and became a consultant in traffic, environment and sustainable transportation. [I have] worked more with heavy vehicles too, logistics […] and some energy issues […], logistics […], fuels […] vehicle fleets […]. Came back here after six years and was hired as a project leader for urban logistics. […] Air quality issues, attached to environmental quality norms, which we’ve faced some problems with…

With a variety of academic backgrounds, employees can solve many problems in the daily humdrum of municipality work, and the administration can make use of generic knowledge among its staff. Academic training provides the employees with something other than specific skills and detailed knowledge. This “something” was reported as more of a “general way of thinking” or a certain ”take” on things in daily work rather than as a specific and applicable expertise related to the concept of proficiency [30]. When asked about what parts of her education (in an environmental discipline) made a difference to the quality of her daily work, one of the respondents reported, slightly hesitantly:

- Well, I don’t know... It’s been a while since I was a student, now it’s more… You lay some sort of basis, but then it’s due to these years when you’ve been working with a variety of issues which I feel have formed my basis.

- A professional experience?

- Yes, yes, precisely, to be able to review the material, to grasp [the content of] reports and other things. Maybe one thing I’ve brought with me [from higher education] is to sort out reports which don’t seem robust or to be able to draw conclusions that “this study is much too small for making this kind of generalisation”. So I guess one brings that, to decide: ”how can I use this report?”. ”How can I use this material”? Does it really say anything [relevant] or not?”

Another respondent, when asked what use she had had of her education, said that “no, not specifically–I somehow slid off the main track of my education”. She also reported that her knowledge about physical planning and GIS was useful, though she mostly had learnt this in her current position. All the respondents, except for two, reported having drifted from their original education. One respondent stood out, as the highly specialised work she was performing within her position was closely related to her education. This respondent also spoke of the scientific minutiae, measurements, and results in her work with more detail than the other respondents. Several of the respondents reported that their professional lives had removed them from academia in general, which some of them regretted, but also saw this as a natural process when entering the job market. With “academia”, interviewees seemed to indicate social circles within the universities as well as the knowledge production happening there. Disciplinary background, thus, was not determining professional positions or practices, but might still be influential in personal networks. Several of our respondents, though, as we will see below, did regret losing contact with academia. 

### 3.2. Hard Data Produced within the Municipal Sphere

Respondents seem to work between, on one hand, what they call “hard data” or ”hard facts”—i.e., information meant to provide them with a foundation for optimal decisions on what to do—and, on the other hand, the “soft”, human, or practical dimension—the realm of the doable. This dichotomy could be understood in the concepts presented by Oepen, where data is the “information”, while what is possible to achieve professionally is the “action” [35]. The data used, though, does not mainly come fresh from research institutions or research publications. Instead, a lot of the research is conducted within the municipality itself, related to what Lundgren and Sundqvist call facts [30]. Air pollution in the city is measured by the Environmental Office, while the noise, also caused by traffic, is measured—and abated—by the Traffic Office. These measurement places are often based on prior knowledge of a problem, where previous measurements have shown levels to be too high in relation to regulations. The results of these measurements are considered “hard facts” or information. Respondents seemed to be content that issues could be handled and then evaluated according to their own available data (in this case, traffic noise):

- Well, in all single issues, we [the Traffic Office] can do measurements, when we use vibrations. We make a pre-and after measurement and we’ll know [if the mitigation worked]. Noise is harder to measure [than air pollution], but then we’ll for example measure the sound absorption of windows, and a new window is put there, and we know it became less noisy. So, in single cases, it can be done.

The civil servants also report that there are conflicts between the actual issues on their table, which might be about exhausts or traffic noise on a specific street, and the “larger” picture, the structural level which municipal environmental plans often focus on:

- When it comes to the more… what should I call it? [general goals] Like for example ”municipal [public transportation] commuting must increase at the cost of private car traffic”–things like that are not easy [to measure], as it [changes] happens over a row of many years. In those cases, one can measure traffic, to know if it actually changed. Or the fact that we have to apply the Plan for Traffic and Mobility in our everyday work – it’s not an easy thing to measure. Or when we have to do campaigns to influence the behaviour of our citizens […] But then there’s the greater noise picture, actually a mapping done every five years. It’s supposed to give us a picture of how our work is going in the right or the wrong direction.

Another respondent witnessed how he’d gone through a transition from “soft” to “hard” aspects of his professional life, and ended up stating that traffic planning is a “hard” sector:

- I had to work harder getting good grades in science, with mathematics and that kind of subjects. And this is what I’ve worked with a lot […]. A lot of emission estimations and statistics and development of methods, not only qualitatively but also quantitively. So it’s been a journey, and eventually, one lands in some kind of ”hard”… – well, it is difficult, this is a ”hard fact” sector.

Information regarding air quality and air pollution abatement fits into the category of “hard data”, even though the respondents report sometimes wishing for a more holistic approach. To obtain acceptance for their actions among policy makers, they need numbers showing effects. One of the civil servants, who returned to the quality of data produced within the municipality itself, emphasised the importance of these as arguments:

- We have [been discussing and applying results] in dialogue with the politicians, the best research results […] like “it is the traffic in the streets of Malmö city that causes the premature deaths of 90–100 persons per year”. That type of results, they are the best arguments. It is [pivotal] that we get access to all these results, and that they are unambiguous. Because there are so many results, ready. We don’t have the time to measure them. And cost calculations are also great, they are in the TROMP [a municipal traffic plan] – if Malmö will keep growing, how should the car fleet look then, so that we don’t burden Malmö more?

Note how this civil servant refers to results and research as something produced within the municipal office itself. The in-house produced data appears to be central to both policy making and practice, taking precedence over research results from outside. Another interviewee responded to the question on where to access information:

- Mostly from the municipal planning and construction atlas, where one can see all ongoing detail plans and one can check noise levels and air quality and many other factors.

- What numbers and data is it [the atlas] based on, do you know that?

- Well, it can differ. The noise maps are from the environmental mapping that we perform every five years, and air [quality] must come from here, from my colleagues [in the Environmental Office]. I don’t know how often they update it. And the other layers… I have a colleague who works on GIS where there is a common map for everything.

It was a recurrent pattern that the civil servants, when referring to data on which to base actions or argue for certain changes with the municipal policy makers, primarily thought of their own sources of information, not of research results from academia. One possible explanation for this might be, as we will see in the following section, that data used in implementation needs to fit into a certain administrative structure and relate to a specific case and situation. 

### 3.3. Administrative Pipelines and Urban Planning

The fact that pollution is the responsibility of one office and noise is the responsibility of another, when preventing the problems is related in practice, made the task of handling information and transferring it to the most efficient action problematic for some of the interviewees:

- How do we cooperate around air quality and noise issues? We try to make them merge. When I got this position, it was put in the same job – it wasn’t like that before – earlier there was one person [on noise and one on pollution]. So, this [assigning both issues to one person] is a way to go. Earlier, there was a Traffic Environment Programme, where they have tried to make them merge. It’s not running anymore. […] The research on air quality and noise also seem very separated, there’s very little joint research. […] If we want to mitigate at a preschool or a school – well, traffic really is the primary source of noise and pollution…

Another respondent also drew the conclusion that the field of noise and pollution mitigation should fare better if civil servants were able to approach things more holistically, as well as with more qualitative aspects:

- But I find myself in a situation where I try - this turns almost existential, but I try to re-evaluate things - I imagine that when I listen more with my heart and my gut-feeling, I actually miss the softer sides of my profession. Actually, I try to change that.

The respondents all reported to live in an administratively fragmented work environment, noting that a more system-oriented approach would make it easier to solve the problems they are facing:

- Do you apply a more holistic perspective or rather look at the details?

- A more holistic perspective. When I get positive feedback, I often hear I’m good at looking at the whole picture […] I don’t think I’ve adapted as much as I’ve kept to my point of departure, but then I’ve had good use of my education, where I worked in transdisciplinary aspects of system analysis.

A more “holistic” approach—i.e., a trans-sectorial and collaborative approach between offices when abating air pollution and noise—was sought after by several of the respondents. One of them reflected, for example, on the fact that noise reduction and air pollution abatement are challenges that would be better handled if there was more focus on the effects of traffic in general, rather than on the separate environmental effects, and that the growing trend for denser cities complicated the work:

- Yes, when it comes to Malmö, it [the traffic] is one of the biggest challenges, and then densification. When it comes to traffic, it might turn out well, but it can also be bad. For the air.

In the case mentioned here, a new shopping mall was planned at the edge of the city centre. When estimating the effects, the Environmental Office expected the mall to cause a surge in traffic. However, according to the interviewee, detailed planning counteracted this development. With the new planning, vehicle traffic (and thus air pollution) decreased, but unfortunately so did local shopping in one of the nearby old main streets of Malmö. 

### 3.4. Everyday Chores/Mill: Routine, Project Administration, and Loss of Organisational Memory 

Work in the municipal administration is mainly based on local routines and experiences. Everyday work is also often project-based, so that funding is allowed for a specific project, or allowed from the politicians during a certain timeframe. There is, as one respondent witnessed, a lack of organisational memory within the municipality organisation itself. This, of course, is a waste of time and resources:

- Something quite problematic in municipal activity is this… lack of re-introduction of already established knowledge [*återföringen av kunskap*]. Sometimes, one might search for something, and you find, just by accident, that this case was investigated ten years ago, too, and there is this and that report on it, so…

With scarce resources and a mainly project-based way of working, a civil servant may not know the details of what has been done before him or her in the same field. By accident, he or she might find old results regarding a similar matter which had been handled years ago. Among the older and more senior civil servants, naturally, there seemed to be a greater sense of “overview”, as years of experience had made the respondents accrete not only knowledge in terms of facts and where to find them but also a mental archive of what had earlier been done in the municipality. 

The project culture, where a specific issue is to be handled by one or several civil servants in a designated timeframe, also seemed to cause some obstacles to the transfer of knowledge—and to the amount of time that could be spent on the issue. One respondent had been responsible for collating an environmental programme for the municipality, but now had to wait for political decisions on whether an updated version should be produced. It was not, by the time of the interview, decided whether the same group of civil servants would be assigned to produce the new programme or how they would do so. 

### 3.5. When Facing the Unknown: Internet and Networks 

The respondents were all asked where they turn to get reliable information about air pollution or noise and how to translate them into practice. They were all serious about the need for contemporary, high-quality research results to “raise the bar” in their practice when arguing for a certain change:

- But what I use scientific results for…, it’s to improve the level of knowledge, motivating why we should do this and that, and why we should spend money on it, why should we build a noise wall here, instead of there, and so on.

Somewhat surprisingly to us, the respondents reported they would start their “research” for a case on a readily available forum: the Internet.

- If I need information, it is most often in a hurry. I Google, look at homepages I know of, often related to traffic issues or environmental issues.

- If you are to start up a new project, and need to find information, how do you go along?

- Well, I Google, I guess.

- Sometimes, I have the time [to do research] – well I guess I start with Googling! And then one sees if something interesting comes up, it might be a study of some kind, and then we can contact those who are responsible.

This last comment about contacting those who are responsible takes us to the next finding, to which degree personal contacts were important to knowledge access and application. All the interviewees mentioned their networks and partnerships with civil servants in other municipalities as central for gathering information. Here are two examples:

- Then there are a lot of [personal] contacts. You know something [comparable work] is going on somewhere…

- What could that be?

- Then you’d call them, like ”You did that, how did it go?” ”Is there anything to share, do you have any report ready?” and often there is, so you can use your network. So networks and the Internet, that’s probably the primary [method of information collection]. […] So I feel it’s the kind of information that’s available, what you find, apparently, but… the scientific [quality] gets kind of lost. I do have contacts at the Lund Technical University and Malmö University, but I rarely use them to find information.

- Sometimes, I have contacted colleagues in the environmental offices in Stockholm and Gothenburg. We know each other, vi meet once a year, the people working with urban planning at the environmental offices, and [I would] ask if they know of something. There we have a little network where we exchange information.

Second to the in-house produced data, online information on related projects was the next step to accessing knowledge. Personal networks provided civil servants with quick information about what was “going on” in the field. These contacts often consisted of colleagues in other municipalities, where one person told the next about new results and sent some links, etc. Contacts with researchers were reported to be scarce. One interviewee expressed that contacts with academia were very good and productive. When giving examples, the contacts consisted of students from university programmes visiting or doing internship projects. In one of these projects, an important finding regarding the particle exhaustion of buses had been made. Another civil servant said that he still had contacts within academia but seldom would use them to access information. In Lundgren’s and Sundqvist′s analysis, familiarity relates to knowledge about the subject matter, which the civil servants build up over time. However, it also relates to encounters or meetings between people and trust built over the years, which may be pivotal for professional efficiency [30].

More abundantly reported as sources of information were the reports produced by the Swedish state or other authorities. These reports are sometimes, but not always, produced by researchers. Instead, they might take the form of extended review articles produced, for example, by the Swedish Environmental Protection Agency. The reports may be of high relevance and quality, but they are discussed and edited in a professional context, aiming more at connecting policy with reality and practice than scrutinizing detail. One civil servant expressed insecurity about where to draw the line between reports and research:

- I sometimes wonder: Where is the line between official reports and research? Because I don’t really know where to draw the line – what is a report and what is research? […] What I consider research, well I’d look more at where they [the papers] come from, if they are from the WHO or from some medical study, or from the Swedish Work Environment Authority. And if there’s some kind of problem description. And then there are innumerable reports, but they are from other sources, maybe The Swedish Transport Administration or other municipalities. They might come from Chalmer’s [University of Technology] or larger projects, so that would be some kind of research, where they try to find solutions and measurements. But there are very few direct solutions, you know ”do this and you’ll solve it”.

The report information was said to be more accessible and available than research results and was also presented in a more accessible form, which is valuable to busy civil servants. 

### 3.6. “I Don’t Take the Time” and ”It Is Not Available”: Obstacles for Access to and Use of Scientific Information

There are two main obstacles to civil servants accessing and applying scientific information in air pollution abatement. Even though (as demonstrated above) the civil servants have a relevant education in their professional field or a neighbouring one, they depict distance to the scientific community as quite a loss in their daily toil. Science and its results are far away, sometimes in their own half-forgotten past. The first obstacle is the lack of time to access research results:

- No, we can’t find [research] – and it’s a big loss, as I see it, something I miss, while I also see that I seldom have had the space, the possibility, time-wise, to do it or I don’t take the time. It might have to do with how you work. […] It has to be quick and good, i.e. you do it as well as possible but as quickly as possible. I which there was more time.

Several times, interviewees pointed out the fact that there is too large a distance between research and their professional reality. Then, there is the lack of digital access—paywalls. The Malmö municipality, like many other municipal administrations, does not pay for access to university research databases or digital journals, where the administration might have been able to make use of the latest and most qualified research results. The civil servants would not be professionally incapable of processing research results but find them unavailable and, if accessible, too complex and detached. In one of the last interviews, the interviewer suggested the general impression that access to research results might be “slightly random” in the municipality, and if the interviewee agreed on this:

- Yes, I agree. Information does come [to our knowledge] but it is not regular or systematic or anything.

- And you don’t have access to search engines and [scientific] publications?

- I don’t even know that. It’s nothing that I have tried, anyways.

This response may give the impression of an uninformed civil servant, but the person in question handled large amounts of advanced information. When there is no formal access, sometimes the Internet and personal contacts seem quicker and more convenient:

- Well yes, informally there are people, rather than homepages. If I get into real trouble and can’t find anything, there are a few people I can think of, whom I know to be very knowledgeable. They also have that research perspective and a somehow deeper contact [with academia]. So, it’s more personal contacts, experts […]. Which I find to be very rare. There are too few connections, so to say, between real life and the academia. Which is fairly obvious.

Generally, the civil servants expressed a sense of having access to sufficient information to do their job, but all of them lamented a lack of time to reach beyond the easily available knowledge. Three of the interviewees mentioned an actor in knowledge dissemination that we, the researchers, had overseen: consultant firms. These were talked about as reliable sources of knowledge, and since consultants were known to have access to research information and were seen as skilled in choosing and packaging results in an easily accessible way, interviewees expressed that they would turn to reports written by consultants if needed. 

### 3.7. The Luxury of Reading and Shrinking Time

A need for easily accessible information is probably a consequence of the lack of time assigned to go into detail in a field of knowledge. The civil servants report that they miss the “luxury” of studying a field in-depth. Reading is something they must “indulge” in rather than a part of the daily work:

- Well, I think […] that there is way too little time for that [reading], generally talking, and that I wish there was more time, and more acceptance from my own side to really just sit down and peacefully process information, which is something I very seldom indulge in.

As for most of office employees, the everyday work of the civil servants interviewed in this study is mostly made up of routine assignments, which they are familiar with and have a lot of professional experience handling. However, as civil servants in the environmental and planning offices, their work also consists of large-scale projects and following up on policy processes. One interviewee, who earlier had said that he would attend seminars and lectures to access new information, related this to a growing department and more stress:

- Yes, administration has increased and it might make the office less efficient when you are more people. Maybe it’s what happens when there are more people working parallel with the same thing, there is always this risk when you grow to big [numerous]. But if you look at the number of sick-leaves, too high work load… well it has grown.

- Do you have any idea how that might be?

- Well, I think […] there is so much information. Everything is available. And… one has a freer role today than before. So there are more possibilities, so if you are supposed to attend more seminars and lectures, and other chores arrive [at your desk], a lot of formal requests come in with a short window to answer, and we produce all these action plans that have to be handled politically – it always takes more time than one might think.

This civil servant stated that an increasing load of unrelated administrative tasks, like filling out forms and time-reports, had fallen on the individual employee over the last decade, which he indicated had decreased the hours available for accessing and processing research results. He then returned to the fact that access to research still could be handled with personal contacts within other municipalities or in academia. 

## 4. Conclusions: Knowledge Access Practices and Obstacles to Research Translation

**Figure 3:**
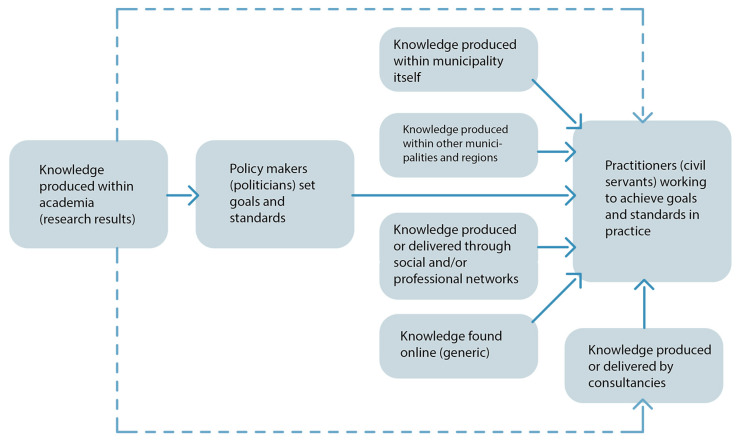
Knowledge access practices found among the interviewed civil servants.

Investigating the translation of research results among the civil servants working with air pollution abatement and noise abatement in Malmö municipality, we found that the interviewees were well-educated and that scientific thinking and praxis remained with them in their professions. They all reported having sufficient information to do their job. We also found a number of knowledge access practices that seemed to provide the civil servants with information in other formats and through other channels than the expected (compare to Figure 1). Firstly, the interviewees referred to knowledge in the form of “hard data” or “facts”. This information, though, would not formally qualify as research results. It consisted of measurements and mappings regarding specific cases of, for example, traffic flows, air pollution, or noise at certain places in Malmö and was produced within the municipal administration itself (see Figure 3). Secondly, interviewees referred to results derived from projects in other municipalities or counties where similar issues had been addressed. Thirdly, to access this form of knowledge, a civil servant may turn to his or her professional network for outcomes, examples, or further readings. Knowledge derived via the social network is relatively accessible and considered trustworthy, since it is provided by people who the civil servants trust. In terms of access practices, the second and third category overlap. The fourth knowledge access practice, when the field of inquiry is new to the civil servant, is “Internet” and “Google” (see Figure 3). A Google search on project-specific keywords gives an insight to what is currently being done in the field anywhere in the world and can work as a quick overview. Since few research findings are directly available through such searches, the results mostly consist of either official reports or examples and are therefore hard to separate from the results derived from the second knowledge access practice, but lack the quality of having been approved by trusted colleagues. A fifth knowledge access practice, though only mentioned by a few of the civil servants, is to request information from consultancy bureaus, who are expected to have more direct access to research results (see Figure 3). One of the civil servants had worked in a consultancy firm, and in this case the knowledge access practice of turning to a social network overlapped with the consultancy knowledge access practice. Scientific results derived from research publications, when mentioned among interviewees, were accessed through social networks too. Only one of the eight interviewees mentioned work in direct contact with researchers and readily referred to research articles.

We encountered a number of routine-related and practical obstacles to access to scientific results, most of them structural and procedural side-effects of how daily work is organised in a Swedish municipality. The first one is lack of time. Civil servants experience an increasing workload related to a project-based and self-administered work culture. Time-consuming activities such as in-depth research and reading are not prioritised. Thus, knowledge has to be accessed in the least time-consuming way. Social networks function as an organic by-pass to access knowledge based on trust and human encounters, as shown in the study by Lundgren and Sundqvist [1,30]. The second obstacle is practical but central: most research is published behind paywalls in journals and research databases, and Malmö municipality does not provide access to these platforms. To access research articles, civil servants have to spend precious time and call on their professional network within academia, if they have one, to provide them with research articles. The third obstacle is that research sometimes is found not to be conducted or presented in ways that are easily translatable into action within the specific municipal context. 

To conclude: despite the self-reported sufficient access to knowledge among the civil servants in Malmö Municipality Environmental Office, up-to-date research results generally do not reach practitioners in an optimal way. Except for in-house produced data, social and professional networks function as primary access channels. The weakness in these knowledge access practices, as is often the case with close-knit networks, is that knowledge risk being reproduced and hence conservative [31] rather than innovative. If accessed, though, a lack of applicability in research results tend to distance civil servants not only from new research findings as such but also from innovative methods and theories within their professional field. With regard to the substantial sums of tax funding put into research, we argue that the lack of efficient routines and structures for the dissemination of research results is a waste of society’s time and money. Insufficient dissemination might delay transitions to a more healthy and sustainable society and ought to be addressed. One way to do this, as we have attempted to do in the ARIEL project, is collaboration between academia and practitioners. With meetings, feedback, and conscious dissemination, research can be produced when and where it is needed to optimise work towards a sustainable urban development. Research can answer questions that are asked and package results in a way that is accessible to practitioners. Collaboration improves contacts between researchers and practitioners which facilitates more efficient knowledge access and more dynamic knowledge translation. Though this project focuses on air pollution abatement in a specific setting, we point to the fact that there are similar obstacles in many other fields of administration for building a sustainable society where research could be better translated, accessed, and applied.
